# Preparation and Properties of Wall Coatings with Calcined Shell Powder as Fillers

**DOI:** 10.3390/ma12142213

**Published:** 2019-07-10

**Authors:** Chun Chen, Yongchao Liu, Qiang Tang, Peigen Zhang, Yamei Zhang, Zhengming Sun

**Affiliations:** 1 Jiangsu Key Laboratory of Construction Materials, School of Materials Science and Engineering, Southeast University, Nanjing 211189, China; 2 Jiangsu Key Laboratory of Advanced Metallic Materials, School of Materials Science and Engineering, Southeast University, Nanjing 211189, China

**Keywords:** shell powder, calcination, coating, reflectance

## Abstract

Using as thermal reflection coating fillers is a significant recycle method for the largely available by-product of shell powders in aquaculture. However, the organics in the shell powder harm its reflection ability. To enhance the thermal reflection performance of the shell powder filled coatings, in this work, the calcined shell powders were used to fill coatings, and the performance of the coatings filled by the calcined shell powders under different temperatures was comparably investigated. Experimental results indicate that after calcination at 400 °C, the organics in the shell powders are removed, whereas the crystal structure of the calcium carbonate is maintained and its particles are refined, leading to an increase in its reflectance. Calcination at temperatures higher than 400 °C deteriorates the properties of the shell powder, due to the sintering of the calcium carbonate particles. The coatings filled by shell powder calcinated at 400 °C deliver the best cooling effect and comparable scouring resistance.

## 1. Introduction

In summer of tropics and other hot areas, the external surface temperature of constructions is able to reach 40 °C to 50 °C, and the temperature of the metal surface is even higher because of the intense solar radiation [1]. Air conditioning is used heavily to keep the building space comfortable, which in turn increases the building’s energy consumption. Statistically, temperature controlling in buildings accounts for more than 20% of the total global energy consumption, and this ratio is still increasing [2]. By reflecting the solar radiation, thermal reflection coatings can effectively prevent the surface temperature from soaring, and, thus, improve living environment saving energy [3,4]. Among the major components of thermal reflection coatings, the fillers play an important role in reflecting sunlight and therefore are important for the coatings’ thermal reflection performance [5]. There are several types of fillers, including talc powder [6,7], hollow glass microspheres [8] and metal oxides [4,9,10,11,12]. The spectral optical properties and thermodynamic performance of nano-ceramic coatings receive intensive discussions [13,14,15]; although the complete agreement has not been found yet about the exact mechanism of their insulating effect, it is plausible that their insulating effect comes from a relatively high surface heat transfer resistance [13,16]. Among the nano-ceramic thermal reflection coatings, nano-sized calcium carbonate is the most widely used insulating fillers. Therefore, the thermal reflection coatings filled with nano-sized calcium carbonate have been well investigated [17,18,19,20]. However, the nano-sized calcium carbonate is expensive, due to its complex preparation process [21,22]. On the other hand, the aquaculture industry is developing vigorously, bringing a huge number of by-products of the shells. Landfilling has been the main disposing method, but it causes serious pollution to the environment [23,24]. As a matter of fact, there would be other ways to take advantage of the by-product, as its main component is calcium carbonate, containing minor organics [25,26]. Therefore, replacing nano-sized calcium carbonate with shell powder as a filler for coatings will inevitably achieve huge economic and social benefits. For example, Rujitanapanich et al. synthesized valuable hydroxyapatite powder from oyster shell via precipitation [27]; Santhosh et al. synthesized nano-sized hydroxyapatite (HA) by a wet chemical reaction route using powdered sea shells as raw materials and investigated the thermal stability of as-synthesized HA [28]; seashells were also used as catalyst support by Rostami-Vartooni et al., who immobilized silver nanoparticles on the surface of seashell via a green and mild procedure [29]. These examples demonstrated the possibility for the high-value added utilization of the large-scale by-product.

In our prior work, we had shown it is feasible to use shell powder as fillers of heat reflecting coatings, and the particle size of shell powder has a significant influence on the heat insulating performance of the final coatings [30]. However, the reflectance of shell powder is evidently lower than that of chemically pure calcium carbonate with the same particle size. The spectral reflectance of chemically pure calcium carbonate (9 μm) is 0.926, and that of the shell powder is only 0.848, which may be due to the presence of organics in the shell powder [31]. Therefore, removing the organics in shell powders would be helpful to improve the reflectance of it, and, thus, enhance the cooling performance of the as-filled coatings by reducing the wall gaining energy from the solar radiation.

In this work, to improve the spectral reflectance of the coatings, organic-removing treatment for the shell powder filler was carried out by calcining. The effects of different calcination temperatures on the compositions and the properties of shell powders are discussed, and the properties of the heat reflection coatings filled with the calcined shell powders are investigated.

## 2. Experimental

### 2.1. Raw Materials

The calcium carbonate content of the as-received shell powder used in this research is over 95 wt%, and the average particle size is 3.42 μm, as examined in our previous work [31]; after calcination, the shell powder-derived calcium carbonate was used as fillers in this research. Fatty acid, alcohol and aliphatic alcohol ether were used as a dispersant, antifoaming agent and film-forming agent, respectively. Other materials employed in this research include hollow glass microspheres filler (~30 μm), waterborne fluorocarbon (solid content of 46 to 48 wt% as a film-forming emulsion) and titanium dioxide pigment (~300 nm).

### 2.2. Sample Preparation

**Figure 1:**
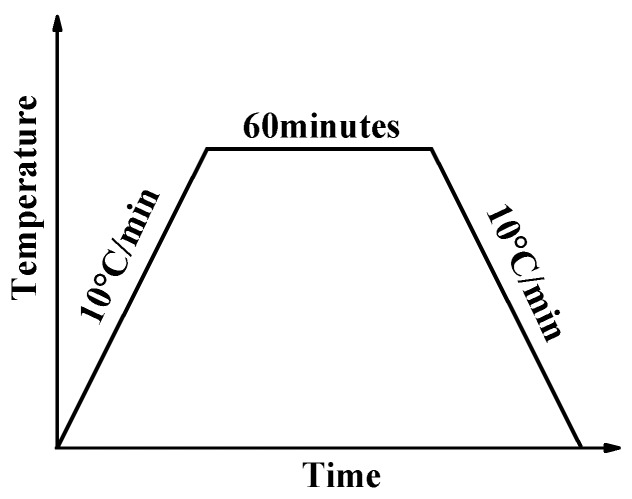
The calcination regime of shell powder.

To remove the organics contained in the shell powders, calcination was carried out in an oven in the air at 100 °C, 200 °C, 300 °C, 400 °C, 500 °C, 600 °C, 700 °C, and 800 °C, respectively. As shown in Figure 1, the temperature of the oven was first increased from room temperature to a specified temperature at 10 °C/min, then held for 60 min at the temperature, and finally cooled to room temperature.

Table 1 shows the recipe of the thermal reflection coatings. Firstly, according to the values contained in Table 1, the shell powder was mixed with water, antifoaming agent and dispersant by stirring at 1000 r/m for 5 min at room temperature. Then, the titanium dioxide, emulsion and film-forming agent were added and mixed for 10 min at 1000 r/m. Finally, the hollow microspheres were added and stirred for 20 min at 500 r/m. Such prepared slurry was coated uniformly on the surface using film coater.

### 2.3. Testing and Characterization

The phase constitution of the shell powder treated at various temperatures was examined with XRD (Cu-K_α_) (Bruker, Karlsruhe, Germany). The 2-theta scanning range was between 10° and 70°, with a step width of 0.02° and a collection time of 0.2 s per step. The morphology of the calcined shell powders was observed using SEM (FEI, Hillsboro, OR, USA).

The reflectance was tested by the Lavy500 UV/VIS/NIR (Ultraviolet/Visible/Near Infrared) spectrophotometer (VARIAN, Palo Alto, CA, USA) based on Chinese national standard (GB/T 25261-2010). The reflectance of the calcined shell powders and heat reflection coatings were calculated through the following formula:

(1) $$\rho_{s}=\frac{\sum_{i=1}^{n}\rho_{\lambda i}E_{s}(\lambda_{i})\Delta \lambda_{i}}{\sum_{i=1}^{n}E_{s}(\lambda_{i})\Delta \lambda_{i}},$$

where $\rho_{s}$ is the reflectance of the sample; $\Delta \lambda_{i}$ is the interval of the wavelength, which can be calculated by $\Delta \lambda_{i}=(\lambda_{i+1}-\lambda_{i-1})/2$; $\rho_{\lambda i}$, and $E_{s}\left(\lambda_{i}\right)$ are the reflectance and spectral irradiance respectively when the wavelength is $\lambda_{i}$; and n is the number of testing points in the wavelength range between 400 nm and 2500 nm. 

The Nicolet 5700-type Fourier infrared spectrometer (Bruker, Karlsruhe, Germany) was used to analyze the composition of shell powders before and after calcination. The calcination temperature effect on the shell powder composition was studied by comparing the Fourier transformed infrared spectroscopy (FT-IR) spectra of the shell powders.

**Figure 2:**
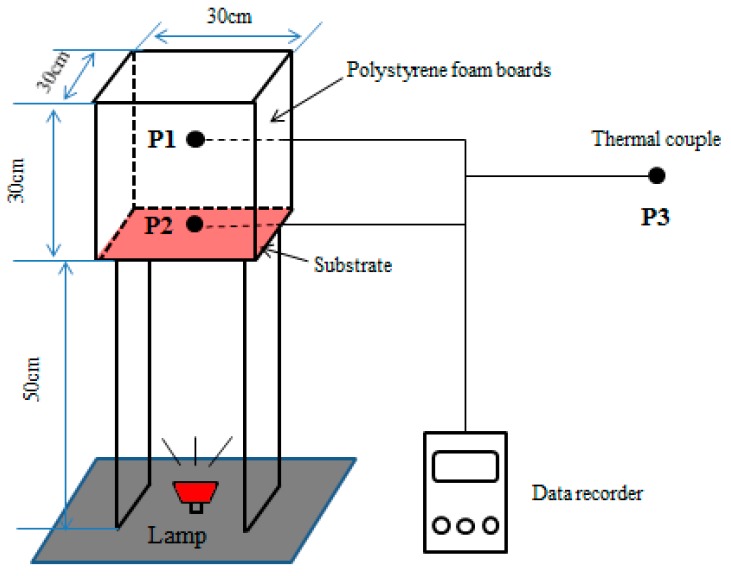
Schematic diagram for testing the heat insulating properties of coatings.

The instrument used for testing thermal insulating properties of coatings is schematically, shown in Figure 2 [32]. An iodine tungsten lamp (500 W) was installed 50 cm underneath the coated specimen, mimicking the solar radiation. A 30 cm × 30 cm × 30 cm box with five walls was made by polystyrene foam board with 2 cm-thick. And in order to shield the environmental influence, the aluminum foil was wrapped in the board. The sixth side facing down was the wall coated by the as-prepared coatings. The walls used in this work were steel plates with 0.2 cm in thickness and cement mortar plates with 1 cm in thickness to simulate the surface of metal structures and the exterior wall of constructions, respectively. Thermocouples were used to sense the temperature variation. The temperatures at P1, P2 and P3 represent the temperature of the inner center of the box, the center of the coating surface and the room not affected by the lamp, respectively. The thermal insulating properties of coatings were assessed by the temperature difference between P1 and P2 irradiated by iodine tungsten lamp for 1 h.

The scrub resistance of coatings was evaluated in accordance with the Chinese National Standard (GB/T 9266-2009). The coating was uniformly coated on the surface of a steel plate with the size of 43 cm × 15 cm × 0.3 cm. Then the steel plate was fixed on the QFS-type scrub tester (Tianjin Jingkelian Material Testing Machine Co., Ltd, Tianjin, China) with a reciprocating brush, and the coating was scrubbed repeatedly until the steel plate was completely exposed. The number of brush reciprocation was used to evaluate the scrub resistance of the coating.

## 3. Results and Discussion

The properties of shell powders treated under different temperatures are investigated, including their composition, morphology evolution, reflectance change upon the calcination temperature. And the thermal performance and the scrub resistance of the as-filled coatings were tested.

### 3.1. Composition of Shell Powders

**Figure 3:**
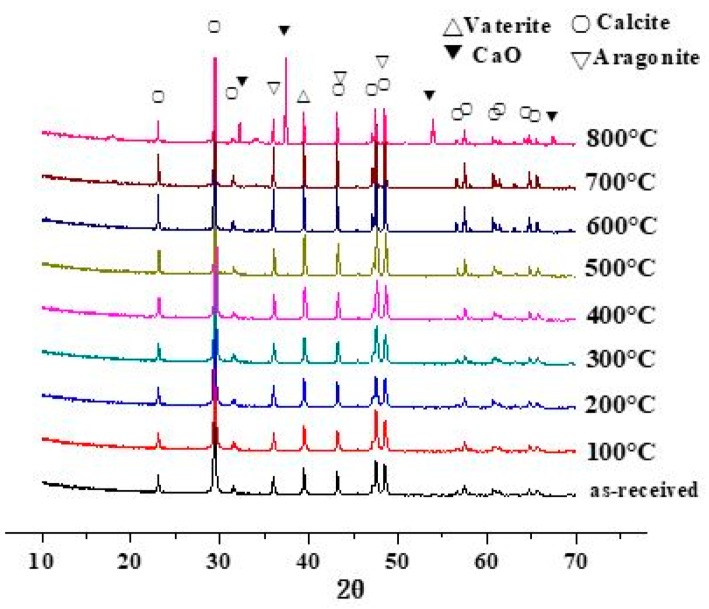
XRD patterns of shell powder calcined at different temperatures.

The phase composition of the shell powders was characterized by XRD. Figure 3 shows the XRD patterns of the shell powders calcined at various temperatures for 1 hour, indicating that the major composition remained after calcining at temperatures below 700 °C consists of calcium carbonate, aragonite and vaterite. Therefore, the shell powder-derived calcium carbonate might replace commercial calcium carbonate where the purity is not a big concern. However, calcining at 800 °C results in calcium oxide, which is the decomposition product of calcium carbonates in the shell powder. By removing the organics, the shell powder can be eco-friendly, and its advantages and environmental friendliness, when used to substitute gypsum binder, was demonstrated by the enhanced mechanical properties, improved thermal performance and remarkable resistance to water absorption by Sakthieswaran et al. [33]. The merits of the recycling of shell powders and the like have been demonstrated when used as an expansive additive in cement mortar [34,35,36].

**Figure 4:**
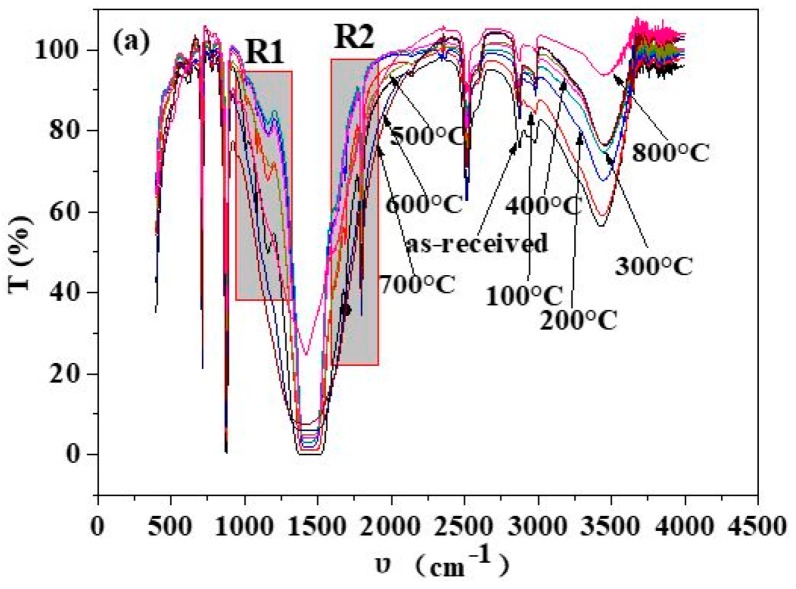

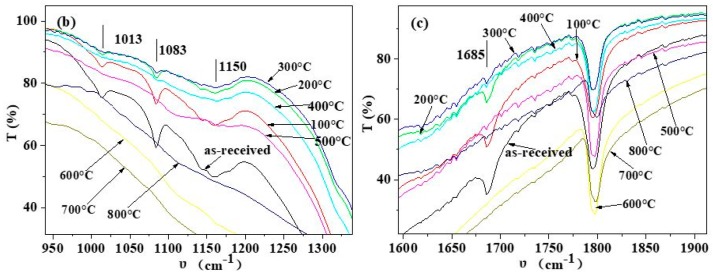
FT-IR spectra of shell powder calcined at different temperatures. (**a**) Full image, (**b**) enlarged the image of zone R1, (**c**) enlarged image of zone R2.

The organics in the shell powders were removed by calcination, and to check the effect of this treatment, IR spectra were recorded and analyzed. As shown in Figure 4, the infrared spectra of shell powders calcined at different temperatures are similar at most of the wave number ranges. In Figure 4b, the peaks at 1013 cm^−1^, 1083 cm^−1^ and 1150 cm^−1^ on the curve for the as-received shell powders are sharp, but intensities of these peaks decrease with increasing calcination temperature and disappear eventually when the temperature reaches to 400 °C and higher. In Figure 4c, the peak at 1685 cm^−1^ also becomes weaker with the increasing calcination temperature and disappears at 400 °C. According to the work of Dauphin et al. [37], the peak at 1083 cm^−1^ can be assigned to the tertiary amide, and it can also be assigned to the symmetrical stretching vibration peak of carbonate ion. The peak at 1013 cm^−1^, 1150 cm^−1^ and 1685 cm^−1^ can be assigned to the C-OH bond, the C=O of fats and amides, respectively. Therefore, the peaks at 1013 cm^−1^, 1083 cm^−1^, 1150 cm^−1^ and 1685 cm^−1^ can be all assigned to organics associated with the shell powder. The content of organics in shell powder gradually decreases with the increasing calcination temperature and disappears eventually, as they decompose at 400 °C and higher temperatures.

### 3.2. Morphologies of Shell Powders

**Figure 5:**
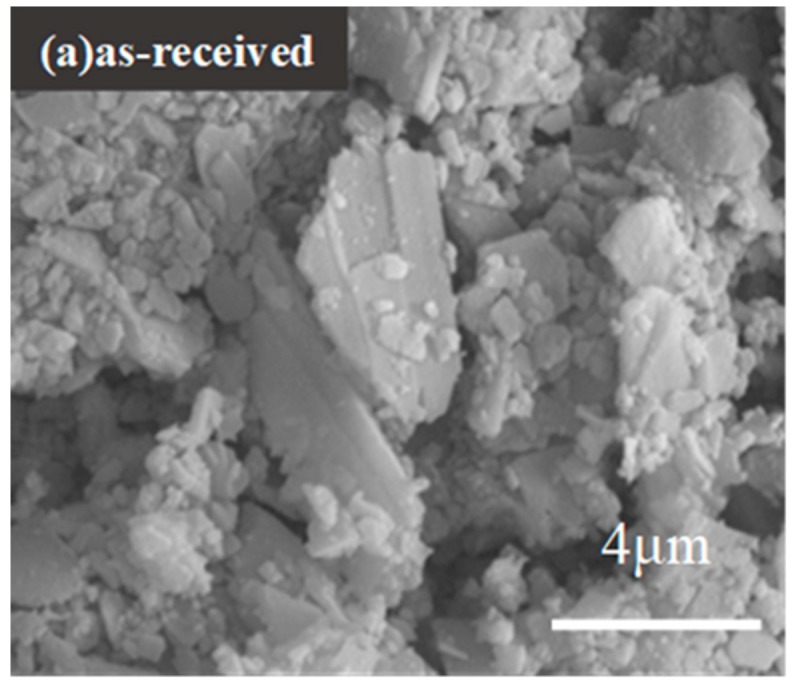

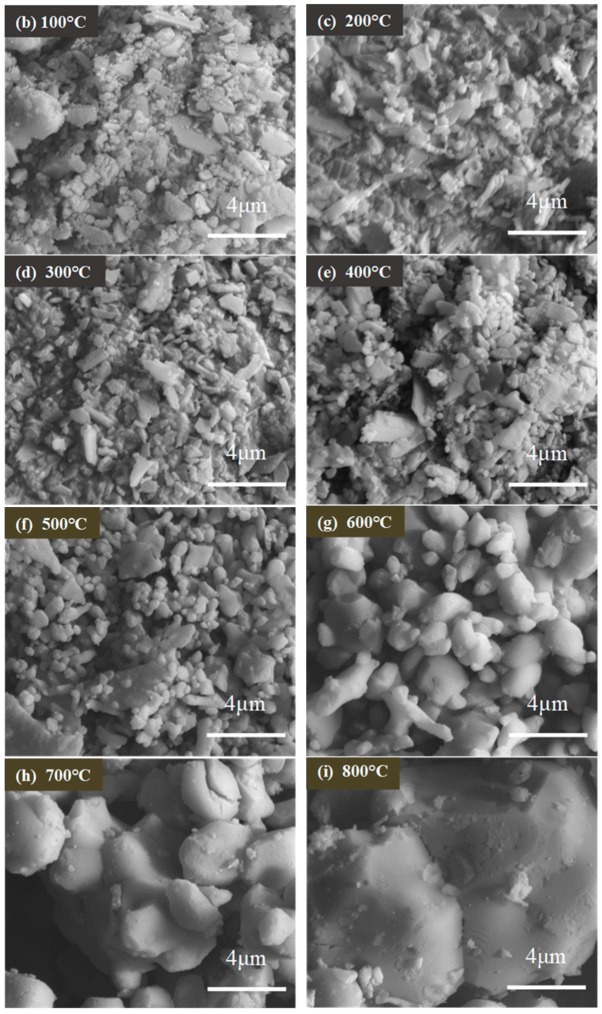
Micrographs of shell powders calcined at different temperatures.

SEM images of the shell powders calcined for 1 hour at different temperatures are shown in Figure 5a–i. The as-received shell particles present distinct layered and angular morphology, Figure 5a. As shown in Figure 5b–e, the morphologies of the shell powders calcined at 100 °C to 400 °C do not change much, though the particle size reduces after being heated under higher temperatures. After being calcined at 500 °C, most shell powder particles change from their original irregular shape to a relatively spherical shape, as shown in Figure 5f–i, and it can be seen that the particle size increases with the further increasing calcination temperature, due to the sintering of particles. Based on the morphology evolution upon the calcination temperature, 400 °C might be a critical temperature for the properties of the calcined shell powder, and, thus, the as-filled thermal reflection coatings, since the shell powder calcined at 400 °C has the finest size and virtually does not contain organics any more.

### 3.3. The Reflectance of the Shell Powders

**Figure 6:**
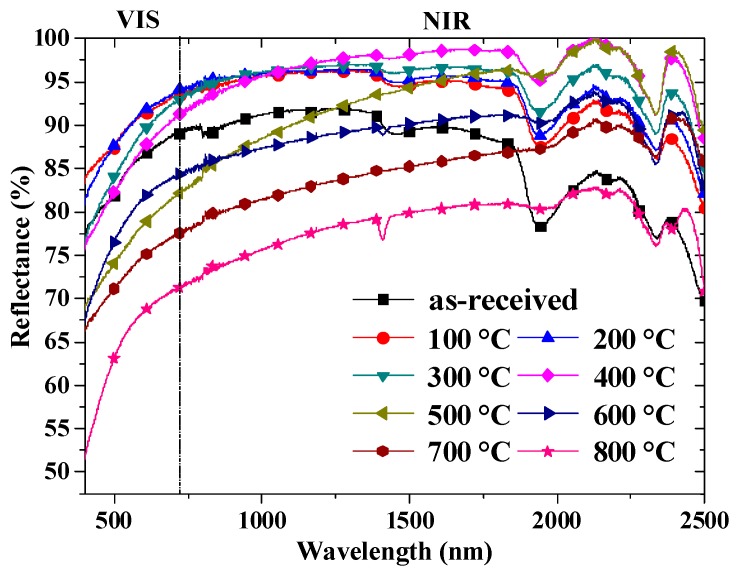
The reflectance of the shell powder calcined at different temperatures.

The reflectance properties of the shell powders calcined at different temperatures are shown in Figure 6. The shell powders all have a higher reflectance in the NIR area than the VIS area regardless of the calcination. But, the reflectance of shell powders after calcination at 100 °C, 200 °C, 300 °C and 400 °C is higher than that of the as-received shell powders. However, the reflectance of shell powders drops when the calcination temperature rises to above 400 °C, in particular in the VIS range. In addition, the shape of reflectance curves of the shell powders calcined above 400 °C is different from those calcined below 400 °C, which can be attributed to the disappearance of organics and the shape change of the powder particles. Since there are some intersections between the various reflectance curves, specific reflectance values are calculated based on the data in Figure 6 according to the Chinese national standard (GB/T 25261-2010), and the results are shown in Table 2. The highest total reflectance, 0.956, is achieved for the shell powder calcined at 400 °C, which is 12.74% higher than that of as-received shell powder. This phenomenon demonstrates that the removal of organics from shell powder can effectively increase the reflectance of shell powders. Considering the results from FT-IR and SEM observation, the organics in the shell powder calcined at 400 °C are removed completely, and the shell particles are refined into small regular spheres which contributes to good dispersion as preparing the coating slurry. When the calcination temperature goes higher than 400 °C, the sintering of particles leads to the increase in particle size, as shown in Figure 5f–i, which results in the decline of available surface area for reflecting.

### 3.4. Thermal Performances of Coatings

According to the above results, the shell powders calcined at 400 °C exhibits the highest reflectance. Since the shell powders were obviously sintered at temperatures higher than 400 °C, they are excluded in the following part of this work.

Table 1 shows the compositions of coatings filled with the shell powders calcined at different temperatures. The coatings were applied on aluminum plates to form a film of ~100 μm in thickness. The reflectance of the coatings was calculated by the same method as that of shell powder, and the results are shown in Table 3. It is clear that the total reflectance of the coatings increases firstly and then decreases with the increasing calcination temperature. When the coating is filled with shell powders calcined at 400 °C, its total reflectance reaches the maximum of 0.796, which is 10.4% higher than 0.721 achieved by the coating filled with as-received shell powders. The enhanced reflectance might be attributed to the reduced size of the shell powder-derived calcium carbonate and the removal of the organics. However, the total reflectance of coatings filled with shell powders calcined at temperatures higher than 400 °C is even lower than that of the coatings filled with as-received shell powders. This might be attributed to the interface area reduction when the particle size becomes larger, due to sintering as the calcination temperature is higher than 400 °C, shown in Figure 5.

**Figure 7:**
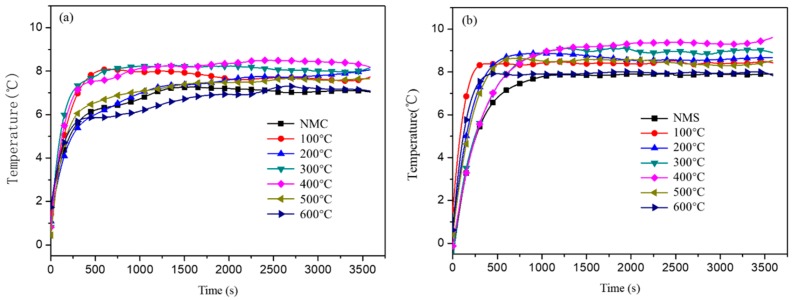
The temperature difference between inside and outside of plates coated with calcined shell powder as fillers (**a**) cement board, (**b**) steel plate. NMC and NMS represent cement board and steel plate coated with the coating filled by the as-received shell powder.

The high reflectance of the coating filled by calcined shell powder is supposed to be beneficial to its thermal reflection performance, since it can reflect more solar radiation away from the wall, minimizing the heat gains on the outer surface of the wall and consequently decreasing the temperature rise inside buildings. That means that the coating filled by shell powders calcined at 400 °C may demonstrate better cooling performance just by virtue of good reflectance even though its thermal conductivity or thermal emissivity may not be obviously changed. To compare the performance of the coatings filled by the shell powders calcined at different temperatures, we measured the cooling effect of the coatings. As shown in Figure 7, the temperature difference between the inside and outside of the box increases significantly in the first 10 min, but later the changes of the difference are not obvious; finally, the temperature difference stabilizes after around 20 min. The interior of cement mortar boards contains a large number of pores, but the structure of steel plates is more homogeneous. Therefore, when using a steel plate as a substrate, the time required for the temperature difference to reach a constant value is shorter than that using cement mortar plate as the substrate. The thermal reflection coatings prepared with shell powders calcined at 400 °C exhibits the biggest temperature difference regardless of the substrate. This means, compared with other calcination temperatures, the shell powder calcined at 400 °C can deliver a better cooling effect when used as filler for coating. The cooling effect of the coatings (Figure 7) agrees with the order of the reflectance ability of the calcined shell powders (Figure 6), suggesting that increasing the reflectance of the fillers can effectively boost the cooling effect of the coatings by minimizing the heat gains from solar radiation.

**Figure 8:**
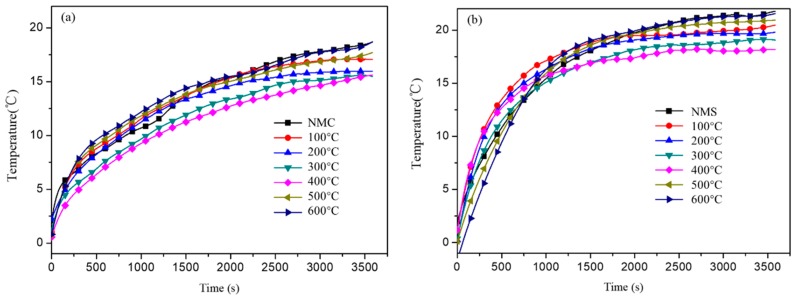
The external temperature rise of coatings with calcined shell powder (**a**) cement board, (**b**) steel plate. NMC and NMS represent coating with as-received shell powder.

The temperature increase of the coatings under radiation is another indicator of their reflectance, i.e., a smaller increase in temperature implies a better reflectance. As shown in Figure 8, the outer surface temperature of coatings keeps increasing even after 60 min on the cement mortar substrate, while the outer surface temperature of coatings on steel plate is almost constant after around 40 min. This can be explained by the fact that the steel is a good thermal conductor, while the cement mortar board with a large number of pores is poor at conducting heat. However, regardless of the substrates, the coatings filled with shell powder calcined at 400 °C always give the lowest temperature rise, as shown in Figure 8a,b, which might be explained by the good reflectance of enlarged interface area and the enhanced phonon scattering effect of fine particle size of the shell powders.

When coated on the cement mortar board and steel plate, the coating filled by shell powder calcined at 400 °C can produce a temperature difference of +8.4 °C and +9.3 °C (Figure 7a,b, 400 °C), +1.2 °C and +1.5 °C higher than that of the coating filled by the as-received shell powder (Figure 7, NMC and NMS), respectively; and the temperature rise is +15.5 °C and +18.2 °C (Figure 8a,b, 400 °C), +3.2 °C and +3.1 °C lower than that of coating filled by the as-received shell powder (Figure 8, NMC and NMS), respectively. All these measurement results demonstrate that 400 °C is the optimal calcination temperature to remove the organics in the shell powders and the as-calcined shell powders have a great potential to be used fillers to be practically used in cooling coatings.

### 3.5. Scrub Resistance of the Coatings

**Figure 9:**
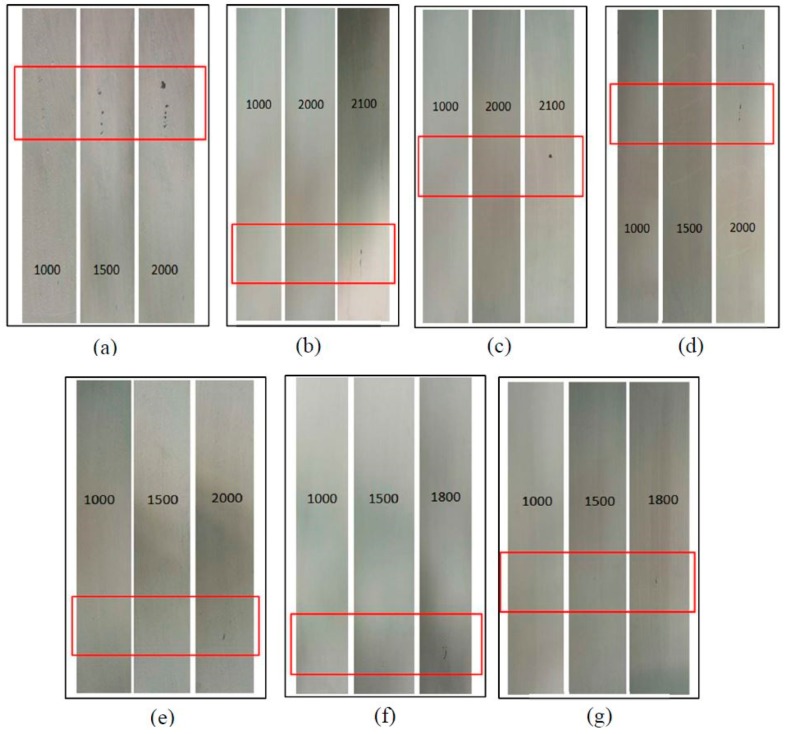
The scrub resistance of coatings with shell powder calcined at different temperature. (**a**) as-received; (**b**) 100 °C; (**c**) 200 °C; (**d**) 300 °C; (**e**) 400 °C; (**f**) 500 °C; (**g**) 600 °C.

The scrub resistances of thermal reflection coatings prepared with shell powders calcined at different temperatures are presented in Figure 9. The coatings were scoured for approximately 2000 times. The scrub resistance of the coatings becomes stronger as the calcination temperature ranging from 100 °C to 400 °C, while it deteriorates as the temperature is higher than 400 °C. This can be primarily explained by the shell powder particle size change upon the calcination temperature. The smaller the filler particle size, the better scrub resistance it can render the filled coatings. When calcined at lower temperatures, the organics in the shell powders cannot be completely removed, and, thus, the particle size of the shell powder cannot be sufficiently fined; as the calcination temperature goes up to 400 °C, the organics are completely decomposed, and its particle size is minimized, as shown in Figure 5e. But as the calcination temperature goes higher than 400 °C, the sintering of the calcium carbonate in the shell powders happens, leading the growth of the particles, and, thus, the deterioration of their properties. 

## 4. Conclusions

Aiming at improving the performance of the coatings filled with shell powder, in this work, the effect of the calcination temperature on the properties of the shell powders and their filled coatings were comparatively investigated. The particle size of shell powders decreases with calcination temperature in the range of 100 °C to 400 °C, but it starts to sinter together at higher temperatures above 400 °C. The calcium carbonate in the shell powders maintained itself when calcined at 400 °C, while the organics therein is completely removed. The total reflectance of shell powders calcined at 400 °C reaches the maximum of 0.956, 12.7% higher than that of the as-received shell powders; meanwhile, the coatings filled with the powder calcined under 400 °C have highest total reflectance of 0.796, 10.4% higher than that of coating filled with the as-received shell powders. The coatings filled with shell powders calcined at 400 °C can survive from ~2000 scouring times before failure. All these results show that the optimal treatment temperature for shell powder used as coating filler is 400 °C, and the measured thermal performance demonstrates that the calcined shell powder has potential to be employed as filler to prepare practical coatings. This would be a high-value added disposing route for the aquatic by-product largely available which otherwise would be a huge environmental concern.

## Figures and Tables

**Table 1:** Recipe of the thermal reflection coatings.

Components	Mass/g	Mass Ratio/%
Emulsion	50.0	37.51
Water	30.0	22.51
Calcined shell powder	15.0	11.25
Titanium dioxide	20.0	15.00
Hollow microspheres	5.0	3.75
Antifoaming agent	4.8	3.60
Film-forming agent	2.5	1.88
Dispersant	6.0	4.50

**Table 2:** The reflectance of shell powder calcined at different temperatures.

Temperature (°C)	Total Reflectance	Reflectance in VIS	Reflectance in NIR
Room temperature	0.848	0.812	0.859
100 °C	0.902	0.889	0.912
200 °C	0.910	0.889	0.921
300 °C	0.934	0.856	0.942
400 °C	0.956	0.852	0.978
500 °C	0.903	0.749	0.912
600 °C	0.851	0.756	0.862
700 °C	0.788	0.721	0.801
800 °C	0.742	0.645	0.752

**Table 3:** The reflectance of coatings filled with shell powders calcined at different temperatures.

Temperature (°C)	Total Reflectance	Reflectance in VIS	Reflectance in NIR
RT	0.721	0.741	0.688
100 °C	0.735	0.785	0.701
200 °C	0.756	0.801	0.721
300 °C	0.781	0.839	0.756
400 °C	0.796	0.846	0.765
500 °C	0.665	0.773	0.623
600 °C	0.649	0.739	0.601
